# SF_6_ is a useful expander for post-pneumonectomy syndrome in the long-term course: a case report

**DOI:** 10.1186/s40792-024-01972-0

**Published:** 2024-07-17

**Authors:** Koki Maeda, Nobuhiro Imamura, Keisuke Tabata, Shoichiro Morizono, Takuya Tokunaga, Aya Takeda, Go Kamimura, Oniwa Masashi, Keiko Mizuno, Masaya Aoki, Kazuhiro Ueda

**Affiliations:** 1 Department of General Thoracic Surgery, Kagoshima University Graduate School of Dental and Medical Science, 8-35-1 Sakuragaoka, Kagoshima, 890-8520 Japan; 2 Department of Respiratory Medicine, Kagoshima University Graduate School of Dental and Medical Science, 8-35-1 Sakuragaoka, Kagoshima, 890-8520 Japan

**Keywords:** Post-pneumonectomy syndrome, Sulfur hexafluoride, SF_6_, Lung cancer, Emergency thoracotomy, Late complication of surgery

## Abstract

**Background:**

Post-pneumonectomy syndrome (PPS) is a rare but serious condition that can occur after pneumonectomy. It is characterized by a mediastinal shift towards the vacated hemithorax, which can potentially lead to respiratory failure. The management of PPS poses a clinical challenge, especially in the context of the limited availability of certain therapeutic devices due to regulatory restrictions in Japan.

**Case presentation:**

A 36-year-old female with stage IB non-small cell lung cancer underwent left pneumonectomy. Approximately 2 years later, she developed dyspnea. After consulting with our hospital, subsequent imaging revealed an extreme mediastinal shift causing bronchial obstruction. Emergency thoracotomy and subsequent sulfur hexafluoride (SF_6_) injections were successfully used to manage her condition. Over the course of follow-up, the interval between SF_6_ injections was extended from 3 to 11 months, indicating an improvement in the intrathoracic condition.

**Conclusions:**

This case illustrates the efficacy of SF_6_ gas in treating PPS and in reducing the frequency of medical interventions. SF_6_ gas administration is safe and effective for the treatment of patients with PPS.

## Background

“Pneumonectomy is a disease in itself”, that is well known to thoracic surgeons.

However, in certain cases, complete excision of pulmonary malignancies is necessary. Post-pneumonectomy syndrome (PPS) is a rare pathological entity following pneumonectomy, characterized by an extreme mediastinal shift towards the vacated hemithorax. In this report, we present a case of respiratory failure secondary to PPS. This case not only contributes to our understanding of post-pneumonectomy patient care, but also serves as a critical educational reference.

## Case presentation

**Fig. 1:**
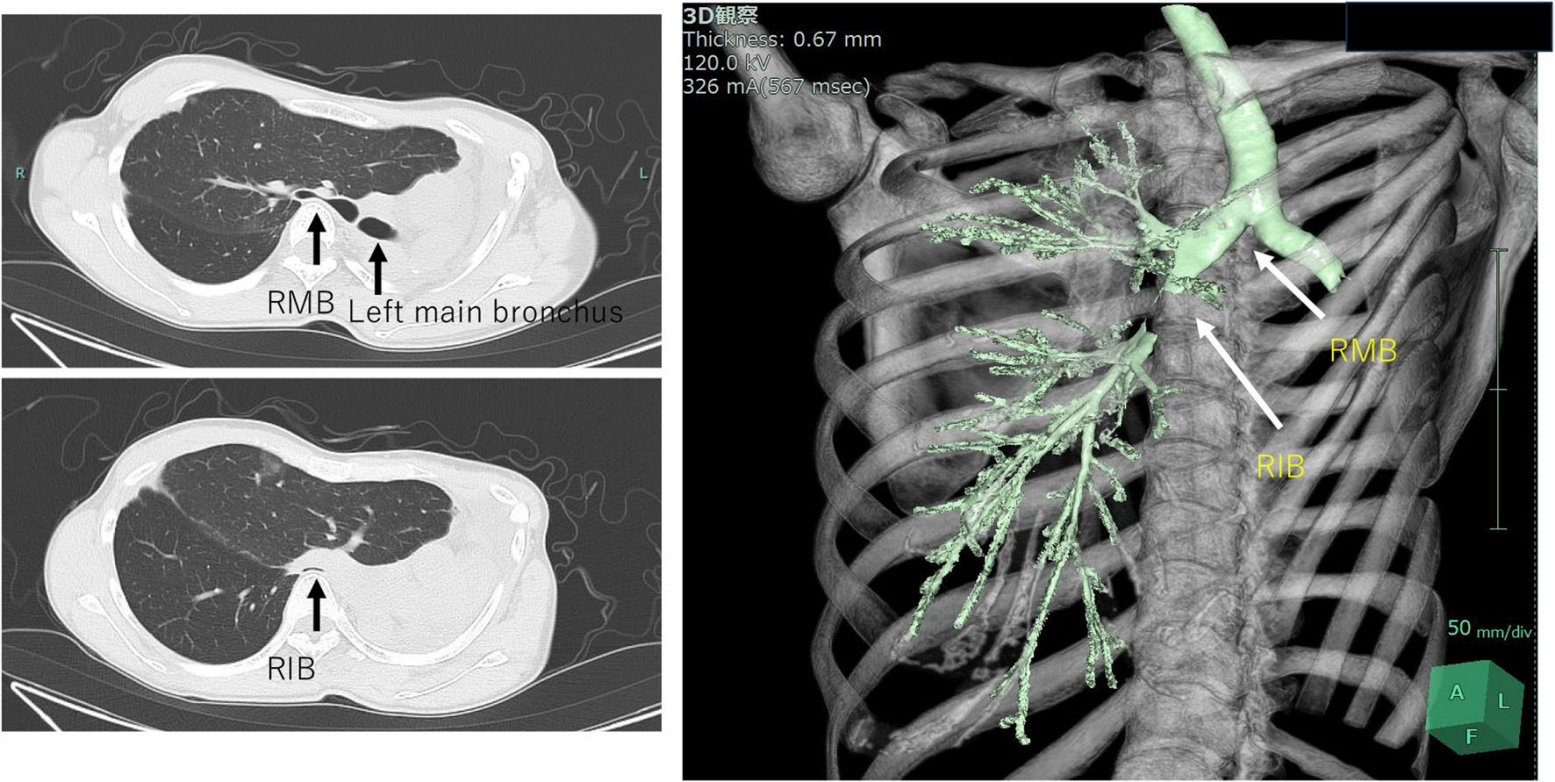
CT scan showed that the right main bronchus (RMB) and right intermediate bronchus (RIB) were compressed by adjacent vertebral bodies, which resulted in constriction. The 3D-CT image provides an overview

**Fig. 2:**
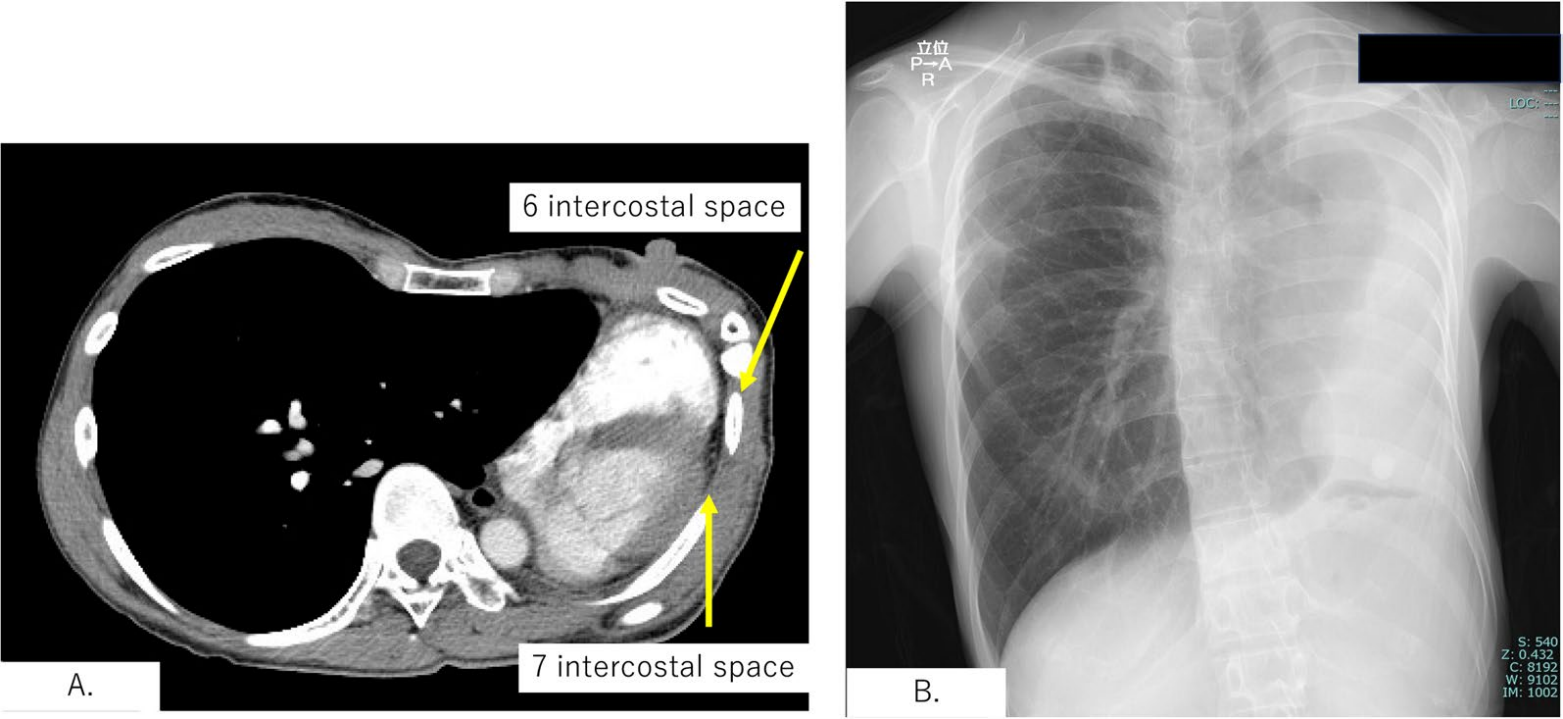
**A** Left thoracic cavity, previously vacant, no longer existed as the pericardial surface had attached to the thoracic pleura. Since the 5th intercostal space was accessed during the left pneumonectomy, we chose the sixth intercostal space for the thoracotomy. **B** X-ray also revealed that the left thoracic cavity had nearly disappeared due to mediastinal shift, with only a small gap between the chest wall and the pericardium anticipated during thoracotomy

**Fig. 3:**
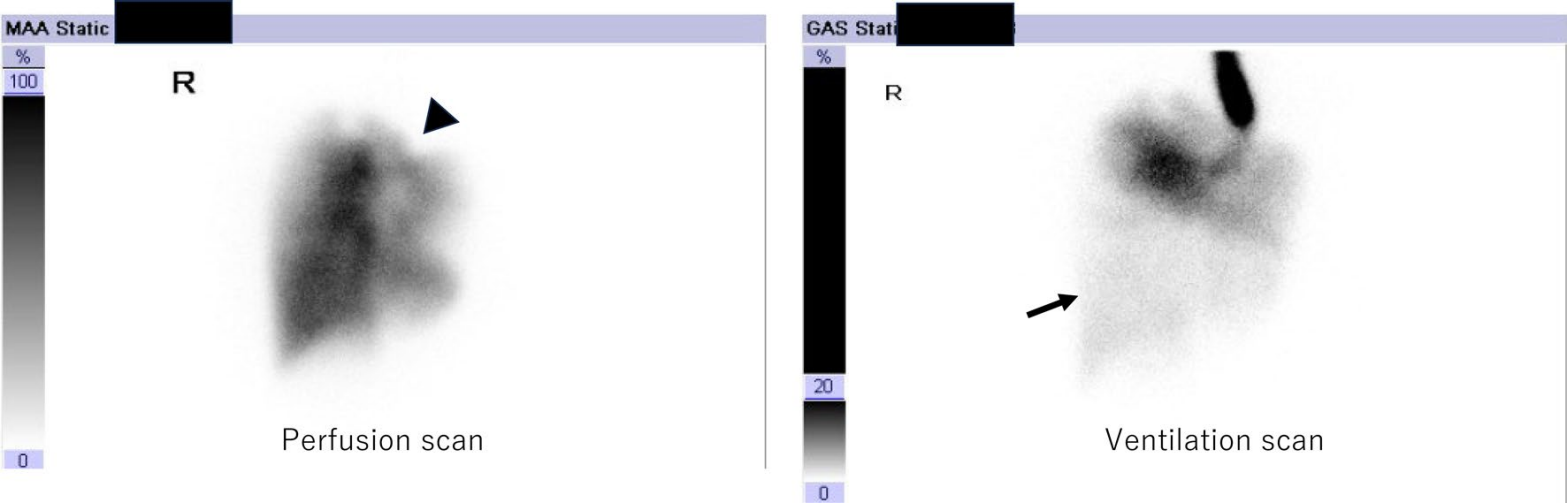
Perfusion scan showing a deposit where PBT was introduced (arrowhead), and ventilation scan showing the complete defect of the right lower lobe (arrow)

A 36-year-old woman underwent left pneumonectomy for non-small cell lung cancer (NSCLC) after chemo-radiotherapy at a community hospital. A pathological examination revealed adenocarcinoma, ypT2aN0M0 stage IB (UICC ver.8). Subsequently, the patient received adjuvant chemotherapy with Uracil Tegafur, followed by surveillance by a pulmonologist. Proton beam therapy (PBT) was administered to the ground glass nodule in the right upper lobe. Two years and 1 month after surgery, she presented with dyspnea. Consequently, she was transferred to our hospital for further evaluation by a pulmologist. The CT scan revealed an extreme mediastinal shift, obstructing the right main bronchus and right lower bronchus (Fig. 1). The left thoracic cavity, previously vacant, no longer existed as the pericardial surface had attached to the parietal pleura. The X-ray also revealed that the left thoracic cavity had nearly disappeared due to mediastinal shift, with only a small gap between the chest wall and the pericardium anticipated during thoracotomy (Fig. 2). A perfusion scan showed deposits where PBT was introduced, and ventilation scanning revealed a complete defect of the right lower lobe (Fig. 3). Based on these results, the respiratory failure was attributed to airway obstruction. Despite the introduction of non-invasive positive pressure ventilation (NPPV) for oxygenation, the patient’s respiratory status deteriorated further. An urgent consultation with our department was requested, and emergency surgery was performed because of her grave respiratory condition. Upon arrival in the operating room, she lost consciousness and exhibited type 1 respiratory failure. Cardiovascular surgeons prepared for veno-venous extracorporeal membrane oxygenation (ECMO) and catheters were placed while anesthesiologists provided respiratory support. She was swiftly positioned in the right lateral decubitus position, and an 8 cm skin incision thoracotomy was performed with utmost caution to avoid cardiac injury. After skin incision, the 6th intercostal muscles were dissected in layers by Metzenbaum scissors, and the parietal pleura was bluntly opened using Langenbeck retractor. Since the 5th intercostal space was accessed during the left pneumonectomy, we chose the 6th intercostal space for the thoracotomy. Her respiratory status promptly improved following the release of air into the left thoracic cavity. Subsequently, 1 L of saline was instilled into the vacant thoracic cavity, and a 12 mm chest tube was inserted not for its conventional use of drainage but exclusively for intrathoracic injection, for which it was clamped.

**Fig. 4:**
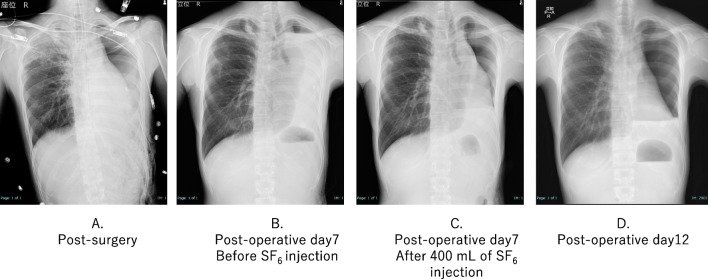
**A** X-ray showed that the mediastinal shift was corrected immediately after the surgery. **B** Air and pleural fluid in the left thoracic cavity were absorbed, leading to progressive mediastinal shift to the left by the seventh postoperative day. **C** 400 mL of SF6 gas was carefully injected into the left thoracic cavity while monitoring the patient's symptoms. **D** Five days after the initial SF6 injection, as the gas expanded according to plan, the volume of the left thoracic cavity increased, resulting in further correction of the mediastinal shift

**Fig. 5:**
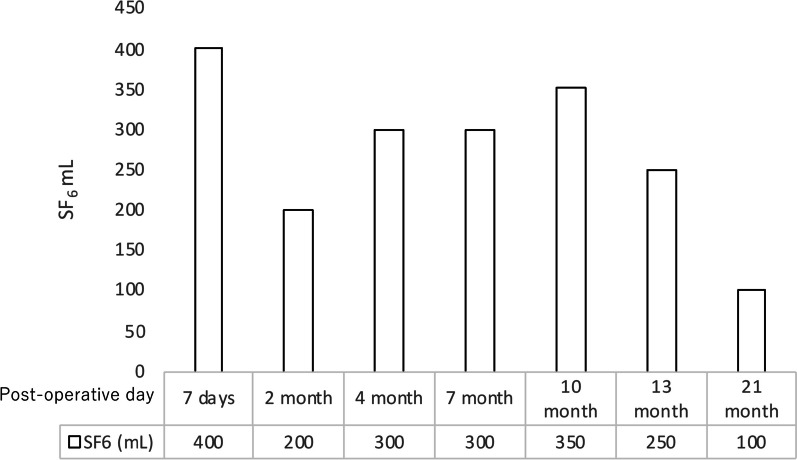
Interval between SF_6_ injections gradually increased from 3 to 6 months during the follow-up period. The most recent follow-up examination occurred at 2 year and 7 month post-surgery, with the interval for SF_6_ injections having been extended up to 11 months

**Fig. 6:**
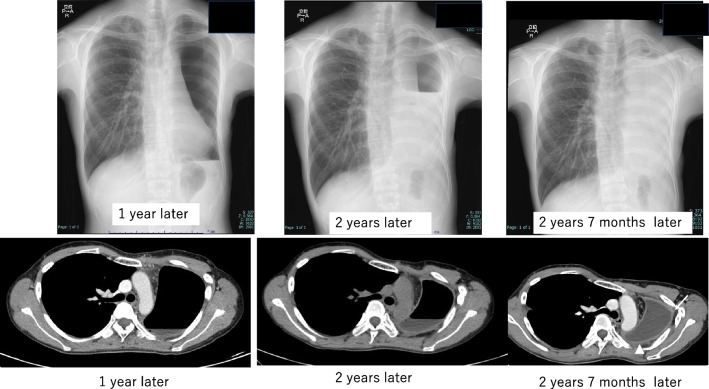
Post-administration course following SF_6_ injection shows progression, characterized by thickening of the left lateral pleura (arrow) and accumulation of pleural effusion over time

Her postoperative recovery was uneventful. However, a gradual midline shift of the mediastinum occurred due to air absorption within the left thoracic cavity. Approximately 1000 mL of saline was introduced via the chest tube to correct the midline shift. To mitigate rapid absorption, we switched from saline to sulfur hexafluoride (SF_6_). SF_6_, which is known for its use as an ocular injection gas in ophthalmic surgery, offers greater stability than saline. The SF_6_ gas was sterilized by transferring it from the cylinder into eight 50 ml syringes through a 0.22 µm filter. In the fluoroscopy suite, we incrementally injected SF_6_ while monitoring the patient's clinical symptoms, continuing until an improvement in mediastinal deviation was noted. The initial injection volume was limited to 400 mL, acknowledging that SF_6_ typically doubles in volume over several days (Fig. 4). Following SF_6_ injection, the patient was safely discharged, and regular weekly to monthly follow-up visits were continued. The interval between SF_6_ injections was gradually increased from 3 to 6 months during the follow-up period. The most recent follow-up examination occurred at 2 year and 7 month post-surgery, with the interval for SF_6_ injections extended up to 11 months (Fig. 5). The examination revealed no evidence of tumor recurrence. CT showed thoracic wall thickening and fluid collection in the left empty cavity (Fig. 6).

## Discussion

In Japan, only 1.8% of lung cancer surgery patients undergo pneumonectomy, making it a relatively uncommon procedure [1]. However, it is important to note that the quality of life of these patients can deteriorate due to complications such as chest pain, arrhythmia, and dyspnea. Bronchial obstruction after post-pneumonectomy remains a rare phenomenon, and there is a paucity of literature addressing this issue [2]. After unilateral lung removal, absorption of thoracic air leads to mediastinal parenchymal shift. The main bronchus and bronchus intermedius were compressed by the pulmonary artery and vertebra. The lack of recent pneumonectomy cases has limited pulmonologists’ exposure to these scenarios. Therefore, this report serves as a valuable reminder of the possibility of bronchial obstruction after pneumonectomy.

The critical condition of the patient warrants preparation of an ECMO system. While this decision may seem controversial, given the obvious need for curative thoracotomy, “stand-by” ECMO was considered essential in this precarious situation based on the patient’s condition and the experience of the medical team.

We opted for an 8 cm skin incision and meticulously dissected the chest wall layer by layer using fine scissors, specifically ‘Metzenbaum scissors’, with utmost care to avoid cardiac injury. CT showed minimal space between the pericardial wall and pleura, and direct attachment between the two structures appeared rare after several months to years of history of mediastinal shift.

Previous studies have reported several articles on the treatment of PPS, including tissue expander implantation [3–6], gas injection [7, 8] and stenting [9]. Unfortunately, tissue expanders for thoracic cavity stabilization are not approved for use in Japan due to insurance regulations, which limits their availability for emergency surgery. In addition, stenting for benign bronchial obstruction is not formally approved in Japan, and the procedure is risky in patients with severe pulmonary conditions. In this case, we employed SF_6_ gas, which is a readily available and straightforward procedure. SF_6_ exhibits twofold expansion within 72–92 h post-injection, subsequently returning to the injected volume within a week [10]. Kimura reported that this patient complained of chest discomfort 3 days after the injection [11]. Nevertheless, to alleviate any chest discomfort arising from gas expansion, 400 mL of SF_6_ was administered under radiographic guidance. It is particularly crucial to administer a conservative amount of SF_6_ into the thoracic cavity during initial administration, anticipating its volume will double in a few days. For subsequent thoracic administrations, the amount administered initially should serve as a reference. It is advisable to carefully elicit feedback from the patient regarding thoracic symptoms experienced in the days following the thoracic administration. As reported by previous investigators, the interval between infusions increases with each subsequent injection. The exact mechanism underlying this phenomenon remains elusive, but it is hypothesized that thickening of the intrapleural wall retards SF_6_ absorption. Indeed, our experience supports this, as we were able to extend the infusion interval from 2 months to more than 11 months, coinciding with gradual intrapleural wall thickening and fluid collection. It is known that C_3_F_8_ is also adapted gas for an ocular injection in ophthalmic surgery which is absorbed more slowly than SF_6_ and remains the thoracic cavity for a long duration. To prevent incidents due to mix-ups, our department of ophthalmology has chosen to exclusively use SF_6_ and has not adopted C_3_F_8_. Although there were plans to purchase and use C_3_F_8_ in this case, it became unnecessary due to the stabilization of the thoracic cavity.

Limitations of using SF_6_ include its status as an off-label application. When explaining to patients, it is necessary to advise against the use of nitrous oxide anesthesia and to avoid high-altitude climbing, air travel and hyperbaric oxygen therapy until the SF_6_ has dissipated. It is essential to obtain patient consent after discussing the off-label use. Following surgeries for post-pneumonectomy syndrome, water and air are relatively quickly absorbed, hence the use of SF_6_ would ideally be within 10 day post-operation. Approval from the hospital’s ethics committee is required for off-label uses, therefore, having a system for expedited review is advisable.

## Conclusions

The diagnosis of PPS is often difficult due to its rarity and the potential for misdiagnosis as other postoperative complications. This case illustrates the efficacy of SF_6_ gas in treating PPS and in reducing the frequency of medical interventions. The use of SF_6_, a gas commonly used in ophthalmic surgery, for intrathoracic insufflation is an innovative approach in thoracic surgery. This demonstrates adaptability in the face of regulatory limitations and product availability. This case provides valuable insights into the treatment options for PPS, highlighting the importance of considering alternative diagnoses of post-pneumonectomy respiratory complications and the necessity of a multidisciplinary approach in emergency thoracic care.

## Data Availability

All related data are included within the article.

## Abbreviations

NPPV: Noninvasive positive pressure ventilation
NSCLC: Non-small cell lung cancer
PBT: Proton beam therapy
PPS: Post-pneumonectomy syndrome
SF_6_: Sulfur hexafluoride
